# The Role of Inflammatory, Anti-Inflammatory, and Regulatory Cytokines in Patients Infected with Cutaneous Leishmaniasis in Amazonas State, Brazil

**DOI:** 10.1155/2014/481750

**Published:** 2014-09-11

**Authors:** Thaís Tibery Espir, Luanda de Paula Figueira, Maricleide de Farias Naiff, Allyson Guimarães da Costa, Marcelo Ramalho-Ortigão, Adriana Malheiro, Antonia Maria Ramos Franco

**Affiliations:** ^1^Coordenação de Pesquisas em Sociedade, Ambiente e Saúde, CSAS, Laboratório de Leishmaniose e Doença de Chagas, Instituto Nacional de Pesquisas da Amazônia (INPA), 69060-001BR, Avenida André Araújo 2.936, 69060-001 Manaus, AM, Brazil; ^2^Programa Multi-Institucional de Pós-Graduação em Biotecnologia (PPGBiotec), Universidade Federal do Amazonas (UFAM), 60077000 Manaus, AM, Brazil; ^3^Departamento de Ensino e Pesquisa, Fundação de Hematologia e Hemoterapia do Amazonas (HEMOAM), 69050002 Manaus, AM, Brazil; ^4^Programa de Pós-Graduação em Imunologia Básica e Aplicada, Universidade Federal do Amazonas (UFAM), 69077-000 Manaus, AM, Brazil; ^5^Department of Entomology, Kansas State University, Manhattan, KS 66506, USA

## Abstract

The authors discuss in this paper the role of inflammatory, anti-inflammatory, and regulatory cytokines in patients infected with different species of *Leishmania* in Amazonas State, Brazil. A comparative analysis was made of serum concentrations of these cytokines in the peripheral blood of 33 patients infected with cutaneous leishmaniasis. The isolates were identified as *Leishmania guyanensis*, *L. naiffi*, and *L. amazonensis*. Most (64%) of the patients were male ranging in age from 18 to 58 years. Protein expression profiles of IL-2, IL-4, IL-6, IL-10, IFN-*γ*, TNF-*α*, and IL-17 cytokines were shown to vary significantly between infected and noninfected (control group) individuals and according to the *Leishmania* species. Infection caused by *L. guyanensis* accounted for 73% of the cases and patients with this parasite also showed higher concentrations of IL-2, IFN-*γ*, IL-4, and IL-17 when compared to infection by *L. amazonensis*. Patients with infection caused by *L. naiffi* showed higher concentration of the cytokines analyzed when compared to uninfected patients; however, there was no statistically significant difference with the other species analyzed.

## 1. Introduction

Leishmaniasis is endemic in several parts of the world, with a global prevalence of over 12 million cases. Divided in two main groups, leishmaniasis can affect the skin (cutaneous leishmaniasis/CL) or internal organs (visceral leishmaniasis/VL). There are 1.5 million new cases of cutaneous leishmaniasis emerging every year [1]. The infection is caused by protozoan parasites of the genus* Leishmania*, transmitted by the bite of the female sand fly vector. Several* Leishmania* species are able to cause a wide spectrum of clinical manifestations of CL, ranging from the mild cutaneous form (localized cutaneous leishmaniasis, LCL) to multiple non ulcerative nodules (diffuse cutaneous leishmaniasis, DCL) and the disfiguring mucosal form (mucocutaneous leishmaniasis, MCL) [2]. In Brazil, American cutaneous leishmaniasis (ACL) is notable for its wide distribution, occurring in all states. In the State of Amazonas, in 2011 alone, 2,230 new cases were notified and the transmission occurred mainly in the cities of Manaus (752 cases), Presidente Figueiredo (213), and Rio Preto da Eva (203) [3]. In the Amazon Basin, seven species of* Leishmania* have been identified as causative agents of ACL. Six such species belong to the subgenus* Viannia*,* Leishmania (V.) braziliensis, L. (V.) guyanensis, L. (V.) lainsoni, L. (V.) naiffi, L. (V.) shawi, *and* L. (V.) lindenbergi*, and one to the subgenus* Leishmania*,* Leishmania (Leishmania) amazonensis *[2, 4]. However, thus far in the State of Amazonas, only* L. guyanensis, L. naiffi, L. braziliensis, and L. amazonensis* have been isolated from humans, with* L. guyanensis* being the predominant species [5, 6].

Clinical manifestations of ACL vary widely, from asymptomatic infections to small lesions limited to or contained within a small area of the skin, to disseminated ulcers and the mutilating mucocutaneous forms [2, 7, 8]. The pathogenesis of ACL is strongly influenced by factors inherent to the host (such as genetic and immune response), the parasite (such as virulence of* Leishmania* species infecting), and vector (as its vectorial capacity). As a result of this interaction between different species of the parasite, the vector, and the mechanisms of the immune response of the vertebrate host, a spectrum of clinical, histopathological, and immunopathological manifestations is observed in humans [2, 9, 10]. Cutaneous leishmaniasis may develop into spontaneous healing or progress to the formation of papules, nodules, plaques, and especially ulcers. Invasion of peripheral lymph nodes and progression to mucosal lesions may also occur. The types of immune response mounted by the individual, crucial for eliminating the parasites, drive such outcomes. Such response, however, is dependent upon host genetic factors and the species of* Leishmania* responsible for infection [2, 8]. Factors associated with the immune response include tumor necrosis factor (TNF); an effective response of natural killers (NK) cells against interleukin-12 (IL-12); and an appropriate production of interferon gamma (INF-*γ*) among other cytokines that might be regulated by the* Leishmania* responsible for infection [11, 12].

Generally, treatment for CL in Brazil is based on antimonial chemotherapy using N-methylglucamine antimoniate (Glucantime, Sanofi-Aventis) [13] and may depend on the clinical presentation and associated comorbidities. Cure rates with antimony are difficult to assess due to the lack of controlled studies of different dosages employed [9]. Moreover, the response to antimonial therapy is likely influenced by the infecting* Leishmania *species [14, 15].

According of the diversity and biological complexity of the parasite, the vector, and the host cellular immune response in ACL, different clinical forms may develop, with* Leishmania *able to direct the production of cytokines and chemokines, cell differentiation, and immune response profile that can lead to cure or persistent disease. Thus, an understanding of the immune response by patients to the infecting* Leishmania* and how effective the current therapy is to the different species of the parasite circulating in the Amazon are of paramount importance for disease control. Hence, the main purpose of this study was to compare levels of inflammatory, anti-inflammatory, and regulatory cytokines in the sera of patients infected by distinct leishmanial parasites and to evaluate the success of the antimonial therapy in 33 patients with ACL treated at a Primary Health Care and the Hospital of Rio Preto da Eva municipality, Amazonas State.

## 2. Materials and Methods

### 2.1. Study Area

The city of Rio Preto da Eva, State of Amazonas, is located near the 80 Km marker on AM-010 Highway (3° 07′06 ′′S, 59° W), 57.5 Km from the state capital, Manaus, and it is part of the Metropolitan Area of Manaus. The vegetation is tropical moist forest upland, with great diversity of species.

### 2.2. Clinical Cases

All cases of suspected ACL were examined at the Hospital Thomé de Medeiros Raposo and at the Basic Health Unit Manoel Rumão in Rio Preto da Eva, Amazonas. Positive diagnosis in each case was made by direct examination of skin fragments obtained by scraping the edge of skin lesions, followed by staining using a Panoptic kit (LB Laborclin) and observation of* Leishmania* amastigotes under optical microscope. Treatment was performed as recommended by the Brazilian Health Ministry: 10–20 mg/Kg/day (15 mg/Kg/day) for 20 days [16]. A skin fragment obtained from the lesion of each patient also was seeded into biphasic NNN blood agar medium [17, 18] and later expanded in liquid Schneider's Drosophila complete medium pH 7.2 (GIBCO-BRL, Gaithersburg, MD) supplemented with 10% heat-inactivated Fetal Calf Serum (iFCS). Following expansion, parasites were prepared for biochemical identification and cryopreservation. Stocks from each parasite species isolated were preserved in liquid nitrogen using 8% glycerol in Schneider's medium containing 30% iFCS. The study was conducted in accordance with the requirements of the National Health Council (resolution 196/96) and was approved by the INPA's ethics committee on research involving human under protocol 193/2008. Patients included in the study were those diagnosed with ACL caused by different species of* Leishmania* and a control group without any symptoms of* Leishmania *infection and no history of previous infection (negative control), matched for sex and age. All study participants signed the informed consent form (ICF) agreeing to participate in the study.

### 2.3. Identification of* Leishmania* Isolates

The strains isolated were typed by multilocus enzyme electrophoresis using eight enzymatic systems: malate dehydrogenase (MDH, EC1.1.1.37), isocitrate dehydrogenase (IDH, EC1.1.1.42) with substrate NAD and NADP, malic enzyme (ME, EC1.1.1.40), glucose-6-phosphate dehydrogenase (G6PDH, EC1.1.1.49), 6-phosphogluconate dehydrogenase (6GPDH, EC1.1.1.44), Aconitate hydratase (ACON, EC4.2.1.3), and hexokinase (HK, EC2.7.1.1) [19]. Species identification was performed by comparing enzyme profiles with reference strains of* Leishmania (Viannia) braziliensis *(MHOM/BR/1975/M2903),* L. (V.) guyanensis *(MHOM/BR/1975/M4147),* L. (V.) naiffi* (MDAS/BR/1979/M5533), and* Leishmania (Leishmania) amazonensis* (IFLA/BR/1967/PH8).

### 2.4. Peripheral Blood Samples

A total of 5 mL of peripheral blood was collected from 33 confirmed cases of ACL as well as from 19 noninfected controls using BD vacutainer tubes (BD Biosciences). Sera were separated by centrifugation at 1,500 ×g for 15 minutes at 4°C and stored at −80°C. All serum samples were tested for infections with HIV (ELISA HIV Tetra, Biotest/Diasorin), HTLV (Murex HTLV-I+II kit, Abbott), and* Trypanosoma cruzi *(ELISA Chagas III, BIOSChile).

### 2.5. Measurement of Cytokine Levels in Sera

Levels of IL-2, TNF-03B1, IFN-03B3, IL-4, IL-6, IL-10, and IL-17 cytokines were compared among the 33 serum samples obtained from patients with confirmed ACL caused either by* L. amazonensis*,* L. naiffi*, or* L. guyanensis* and from unidentified* Leishmania* sp. Cytokine levels were measured using a FACSCanto II flow cytometer (BD Biosciences, San Jose, CA, USA) and the BD Cytometric Bead Array (CBA) Human Inflammatory Cytokine Kit for T_H_1/T_H_2/T_H_17 (BD Biosciences, San Diego, CA, USA). For each cytokine assessed, the concentration (in pg/mL) and the mean fluorescence intensity (MFI) were calculated using the Array-FCAP software (v3.0.1). The results were analyzed using GraphPad Prism (v6.0) and nonparametric tests (Kruskal-Wallis and Mann-Whitney). *P* values <0.05 were considered statistically significant.

## 3. Results

Twenty-four out of the 33 peripheral blood samples were of cases representing primary* Leishmania *infections and nine were collectively referred to as secondary. This latter group was comprised of patients who had either relapse or were infected a second time. In all patients, skin lesions varied in terms of size, appearance, and number of lesions depending on the infecting* Leishmania* species, as shown in Table 1. Nineteen peripheral blood samples were collected from noninfected individuals (control group). All patients also tested negative for HIV, HTLV, and* T. cruzi*.

In the group of patients referred to as secondary, seven (approximately 78%) displayed a single skin lesion, and one patient (approximately 11%) displayed two lesions in the lower limbs. Most (66%) were unable to or did not indicate the type of treatment received at the time of their first infection. Three patients (33%) confirmed previous antimonial treatment received. All nine patients did recall the presence of typical skin lesion characteristic of* Leishmania* infection, which was confirmed by the attending physician at the time.

Infections with* L. guyanensis *accounted for 19 (73%) of the cases and were followed by infections with* L. amazonensis* with 12% (four individuals) and* L. naiffi *with 9% (three cases). In seven patients (representing 21% of the total number of cases) the infecting* Leishmania* species was not identified. Information of clinical and epidemiological details of the 33 ACL cases is summarized in Table 1. Twenty-one (64%) individuals were males and 12 (36%) were females and ranging from 18 to 58 years in age. Although single skin lesions were observed in the majority of cases (24 individuals or 69%), eight skin lesions were observed in a single individual. The estimated time for the evolution of disease (i.e., appearance of skin lesion) varied widely from seven to 180 days (Table 1) and was based on individual patient account. In terms of their occupation, 100% of the patients were indicated to be farmers. Patient information was obtained via a survey answered individually.

### 3.1. Measured Levels of Cytokine in Patient Sera

**Figure 1:**
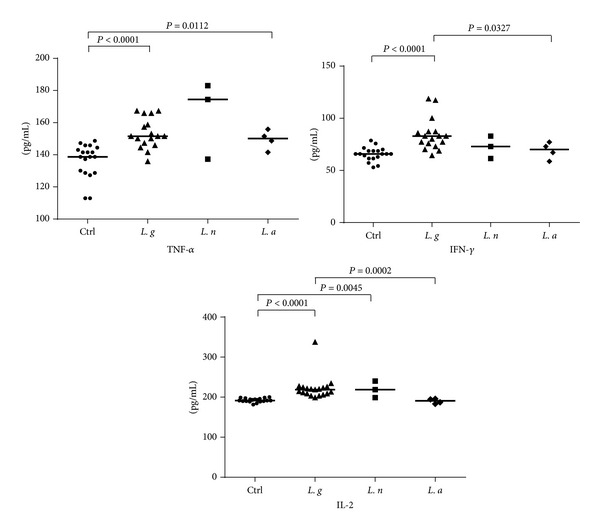
IL-2, TNF-*α*, and IFN-*γ* levels in serum samples from patients infected with different* Leishmania *species and in noninfected control. Cytokine levels (pg/mL) were calculated for each serum sample. Means (horizontal lines) are shown for each group. Ctrl: noninfected controls;* L. g*:* Leishmania guyanensis*;* L. n*:* L*.* naiffi*;* L. a*:* L. amazonensis*.

The levels of inflammatory and T_H_1 profile cytokines IL-2, TNF-*α*, and IFN-*γ* observed in the serum samples from the* Leishmania*-infected patients differed significantly from the noninfected controls, as shown in Figure 1. Similar comparisons between patients infected with different* Leishmania *indicated that infection with* L. guyanensis* led to higher seric levels of both IL-2 (*P* = 0.0002) and IFN-*γ* (*P* = 0.0412) than those from patients infected with* L. amazonensis*. For infections with* L. naiffi*, higher levels of TNF-*α* and IL-4 were consistently detected in comparison to infections with* L. guyanensis *and* L. amazonensis*, though no statistical differences were observed. Higher IFN-*γ* levels also were observed on infection by* L. guyanensis *compared to other species.

**Figure 2:**
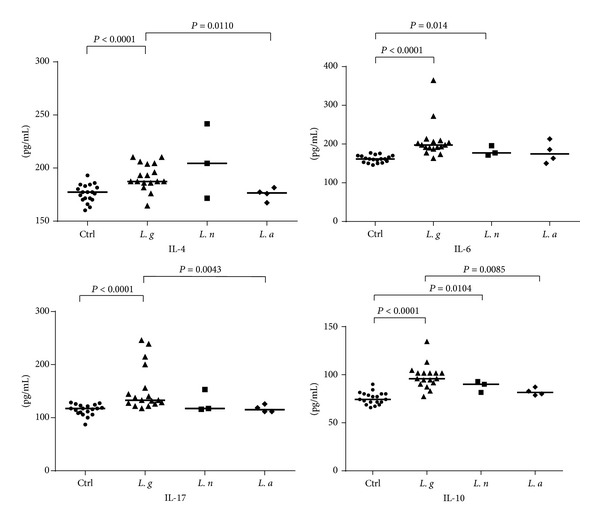
Levels of IL-4, IL-6, IL-10, and IL-17 in serum samples from patients infected with different* Leishmania *species and in noninfected control. Cytokine levels (pg/mL) were calculated for each serum sample. Means (horizontal lines) are shown for each group. Ctrl: noninfected control;* L. g*:* L*.* guyanensis*;* L. n*:* L*.* naiffi*;* L. a*:* L.amazonensis*.

For the regulatory cytokine IL-10, and for the T_H_2 and T_H_17 cytokines such as IL-4, IL-6, and IL-17, our results indicate significant differences when comparing all primary infections with* Leishmania* spp. with noninfected controls. Assessed IL-10 levels were significantly higher in the sera of infected individuals as compared to controls (*P* = 0.0002). Similar observations were made with regard to the levels of IL-4 (*P* = 0.0016), IL-6 (*P* < 0.0001), and of IL-17 (*P* = 0.0002). A significant difference also was observed between the subgroups (i.e., distinct* Leishmania* species) analyzed: higher levels of IL-4 (*P* = 0.0132), IL-10 (*P* = 0.0085), and IL-17 (*P* = 0.0042) were detected in patients with infections caused by* L. guyanensis* than those infected with* L. amazonensis *(Figure 2). Meanwhile, IL-6 levels showed no significant difference in the analysis between the species studied, in spite of being higher in samples from patients infected with* L. guyanensis*.

**Figure 3:**
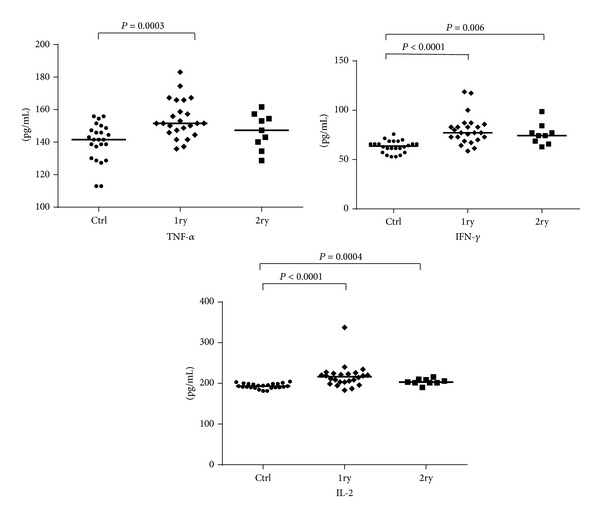
IL-2, TNF-*α*, and IFN-*γ* levels in sera obtained from individuals with primary or secondary* Leishmania* infections and from noninfected controls. Cytokine levels (pg/mL) were calculated for each serum sample. Means (horizontal lines) are shown for each group. Ctrl: noninfected controls; 1ry: primary infections; 2ry: secondary infections.

Seric cytokine levels were generally higher in samples of primary infections as compared with sera obtained from patients with secondary* Leishmania* infection and the control samples. The analysis of T_H_1 cytokine profile revealed significant differences between infected (primary or secondary infections) and noninfected controls for for IL-2 and IFN-*γ* (*P* < 0.0001 for both cytokines) and TNF-*α* (*P* = 0.0009). When comparing the concentrations of these cytokines in the patients with secondary* Leishmania *infection no significant differences between the two groups were observed, despite higher serum levels of IL-2, IFN-*γ*, and TNF-*α* detected in primary infections. Levels of TNF-*α* were lower in secondary than in primary infections but again this difference was not statistically significant (*P* = 0.2100, Figure 3).

**Figure 4:**
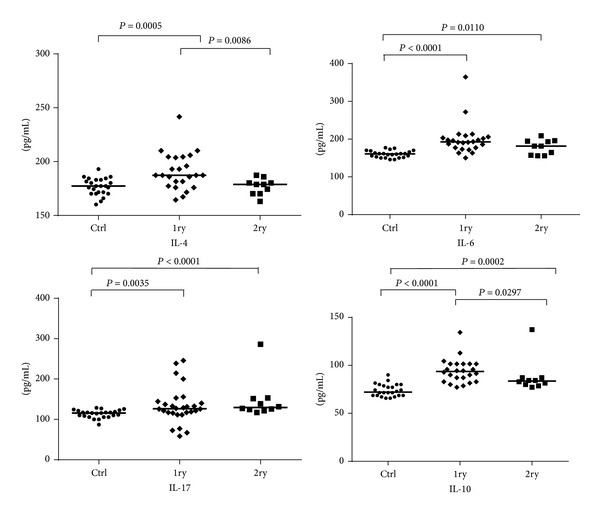
IL-4, IL-6, IL-10, and IL-17 levels in sera obtained from individuals with primary or secondary* Leishmania* infections and from noninfected controls. Cytokine levels (pg/mL) were calculated for each serum sample. Means (horizontal lines) are shown for each group. Ctrl: noninfected controls; 1ry: primary infections; 2ry: secondary infections.

The comparative analysis between the primary and secondary infections and the noninfected controls revealed significant differences for IL-6, IL-17, and IL-10 (Figure 4). For T_H_2 and T_H_17 regulatory cytokines, significant differences in the serum concentrations of IL-4 (*P* = 0.0086) and IL-10 (*P* = 0.0297) were detected between the primary and the secondary infections. For IL6 (0.1200) and IL17 (0.3718) such differences were not statistically significant.

## 4. Discussion

The ACL is characterized by a spectrum of cutaneous manifestations. The classical clinical symptom of the disease is the appearance of an erythematous nodule, which may be single or multiple, usually located at the exposed region in the tegument. In this study we assessed the levels of selected T_H_1, T_H_2, and T_H_17 cytokines in the sera of patients infected with different species of* Leishmania*. Patients had been positively diagnosed with cutaneous leishmaniasis and had not yet started treatment with the antimonial drug Glucantime (intravenous or intramuscular applications of 10–20 mg/kg/day for 20 consecutive days) according to guidelines from the Brazilian Ministry of Health [16].

Cytokines have a central role in the immune response, presenting local and systemic effects [20]. The balance in the production of inflammatory and regulatory cytokines determines the profile of immune response and influence on disease severity [21–23]. In mouse models, resistance to infection or healing is associated with a T_H_1 response that is dependent upon expression of IL-12, IFN-*γ*, and TNF-*α* with production of nitric oxide (NO) [22, 24–26]. In contrast, susceptibility to disease or the inability to control lesion development is associated with a regulatory response with the concomitant production of IL-10 as well as expression of T_H_2 cytokines such as IL-4 and TGF-*β* [27–29].

Cytokines and precursors of T_H_1 profile such as IL-2, IFN-*γ*, and TNF-*α* were present at higher concentrations during the primary* Leishmania* infections when compared to controls. These cytokines stimulate macrophage activity that plays a crucial role in parasite killing [30, 31]. In the comparative analysis between the* Leishmania* species identified in this study, higher concentration of IL-2 and IFN-*γ* in the sera of patients with primary infection, compared to sera from noninfected or those with secondary* Leishmania *infection, was observed. Moreover, levels of IL-2 and IFN-*γ* also were higher in the primary infection group when infected with* L. guyanensis*.

Many cell types, including T cells, dendritic cells, macrophages, and NK cells, produce IFN-*γ*. IFN-*γ* limits the growth of* Leishmania *in human and murine macrophages by stimulating nitric oxide synthesis in macrophages [32]. In murine models, the modulation of local or systemic levels of IFN-*γ* is a critical determinant in the resolution of infection. TNF-*α* produced mainly by macrophages and in concert with IFN-*γ* also leads to induction of nitric oxide synthesis and consequently the death of amastigotes inside macrophages [33]. In 2007, Pinheiro and Rossi-Bergmann [34] indicated that IFN-*γ* is necessary for the control of infection by* L. major *reducing the expansion of T_H_2 cells in experimental infections caused by this species. These authors also suggested that IFN-*γ* role remains distinct in infections with* L. major *or* L. amazonensis *and that in* L. amazonensis* IFN-*γ* is effective in the first week of infection. In addition, IL-10 has an important role with regard to IFN-*γ*, both in terms of expression control as well as induction of IFN-y effectors' function, thus determining the course of* Leishmania* infection [35].

Regarding TNF-*α*, it has been shown that, in the sera of patients with active* L. major *infection or in the sera of patients who received treatment and relapsed, levels of this cytokine do change significantly when compared to a control group [36]. Although that study did not indicate whether these were primary or secondary infections, low levels of TNF-*α* appear to be associated with disease severity during* Leishmania* infection [36]. Here, significantly higher levels of TNF-*α* were detected in primary infections but did not significantly differ between secondary and controls, though were generally lower. Low levels of TNF-*α* are associated with persistence of infection and greater difficulty in resolution of skin lesion [36]. Our results also support this view.

For IL-10, IL-4, IL-6, and IL-17, significant increases in the measurable levels of these cytokines were observed in the infected group compared to controls. In addition, patients with ACL caused by* L. amazonensis* displayed lower levels of sera IL-4 and IL-17 when compared to infection by* L. guyanensis. *IL-17 is known to play a role in inflammation, in autoimmune diseases, and in cancer [37–39]. IL-17 has multiple effects, acting both in the induction of proinflammatory cytokines and in the recruitment and activation of leukocytes. Although its role in CL is not fully understood, this cytokine seems to play an important role in the pathogenesis of the disease [40, 41]. Bacellar et al. [40] observed that IL-17 is produced during infection with* L. braziliensis* and lymphocytes obtained from patients with MCL and CL yielded higher concentrations of IL-17 compared to lymphocytes obtained from uninfected individuals. The presence of T_H_17 cells in lesions of CL/MCL was associated with neutrophils and tissue destruction [40, 42]. Our data point to higher levels of T_H_17 in the sera of patients infected with* L. guyanensis *when compared to the other two species identified (*L*.* amazonensis* and* L. naiffi*). Nevertheless, no significant differences were observed between the sera of primary infections and sera of patients reinfected (secondary infection) with* L. guyanensis*.

IL-6 is produced by many cell types and is involved in acute-phase response in B cell maturation and differentiation of macrophages. IL-6 promotes T_H_2 differentiation and simultaneously inhibits T_H_1 via independent molecular mechanisms. In this work the primary infected patients had high serum concentrations of IL-6 when compared to controls (*P* < 0.0001); the same was not observed in the analysis of patients with secondary infection. In the group of patients with a secondary infection, IL-6 levels were slightly lower than in the primary infection group, but no statistical difference was observed between the two groups. There was no difference in the concentration of IL-6 in the comparison among the three species.

In the murine model, it was demonstrated that the modulation of the immune response by IL-4 produced mainly by T lymphocytes led to the regulation of specific T_H_1 cytokines, such as IFN-*γ* and IL-12 [43]. As suggested by Hurdayal and Brombacher [44], depending on the model utilized, IL-4 appears to be a “double-edgeds word” driving a susceptible T_H_2 response and mediating a T_H_1 response during disease. In our study, patients with primary infection caused by parasites of the genus* Leishmania* (Figure 4) displayed higher levels of IL-4 (*P* = 0.0086) when compared to individuals with secondary infection, suggesting an activation of IL-4 during the first contact with the parasite and its presence in the clinical course of infection caused by various species of* Leishmania*. It was also observed that, in primary infections with* L. amazonensis, *the levels of IL-4 were not as elevated as in infections with* L. guyanensis *or* L. naiffi* when compared with the control group (Figure 2).

The IL-10 plays regulatory role in the immune response, by inhibiting inflammatory mediators and activation of monocytes [45, 46]. It has been demonstrated that the production of this cytokine by CD4^+^ CD25 T cells (Tregs) is associated with persistence of the parasite and reactivation of the disease [47–49]. IL-10 secretion by T_H_1 cells likely is a mechanism that minimizes self-regulatory immunopathology of ACL [50, 51]. We observed lower levels of IL-10 in the secondary infection group of patients in comparison with primary infections. Furthermore, patients infected with* L. guyanensis *had higher IL-10 levels than patients infected with* L. amazonensis* (*P* = 0.0085). These results suggest a profile of mixed T_H_1/T_H_2 response during infection with* L. guyanensis*. Interestingly, we also observed that three individuals infected with* L. guyanensis* (MHOM/BR/11/IM5749; MHOM/BR/11/IM5752; MHOM/BR/11/IM5775) displayed higher IL-10 levels in comparison with other individuals infected with the same* Leishmania *species. Two of these patients with primary infections (MHOM/BR/11/IM5752; MHOM/BR/11/IM5775) had a total of four skin lesions and progression of the disease in about 21 days. It has been shown that, prior to treatment, patients infected with* L. braziliensis* have higher levels of IFN-*γ* and decreased production of IL-10 [52].

Currently, there is no mechanism available to predict whether or in whom the most serious forms of CL may develop [53]. The results presented here suggest that certain cytokines, such as IL-2, IL-4, IL-6, IL-10, IFN-*γ*, TNF-*α*, and IL-17, are involved in the clinical course of human CL in the Amazon and are likely associated with disease outcome, being responsible for either complete healing or remission or on the other hand the development of relapses and more severe forms of the disease.

## Figures and Tables

**Table 1:** Clinical and epidemiological data from patients∗ infected with *Leishmania* spp. from Rio Preto da Eva, State of Amazonas, BR.

Leishmania isolates		Patients data		Infection^3^			
Designations^1^	Species^2^	Sex	Age	Length	Status	Number of lesions	Lesion size
			(years)	(days)			(mm)
MHOM/BR/09/IM5553	*L. naiffi *	M	34	120	P	1	ND
MHOM/BR/09/IM5562	*L. naiffi *	M	26	160	P	2	2 × 2
MHOM/BR/09/IM5584	*L*. *amazonensis *	M	58	45	P	1	8 × 8
MHOM/BR/10/IM5637	*L*. *guyanensis *	F	31	60	P	1	17 × 18
MHOM/BR/10/IM5641	*L. guyanensis *	F	41	150	S	3	21 × 15
MHOM/BR/10/IM5653	*L. guyanensis *	M	18	90	P	1	30 × 20
MHOM/BR/10/IM5657	*L*. *guyanensis *	M	56	37	P	1	19 × 17
MHOM/BR/10/IM5665	*L. naiffi *	M	23	90	P	3	8 × 6
MHOM/BR/10/IM5684	*Leishmania* sp.	M	41	ND	S	1	ND
MHOM/BR/10/IM5690	*L*. *guyanensis *	F	58	60	P	1	17 × 18
MHOM/BR/10/IM5692	*L. guyanensis *	F	30	15	P	2	60 × 60
MHOM/BR/10/IM5694	*L*. *guyanensis *	M	29	14	P	1	20 × 23
MHOM/BR/11/IM5697	*L*. *guyanensis *	M	19	14	P	1	13 × 16
MHOM/BR/11/IM5749	*L*. *guyanensis *	M	38	30	P	1	17 × 14
MHOM/BR/11/IM5752	*L*. *guyanensis *	M	34	21	P	4	11 × 12
MHOM/BR/11/IM5773	*L*. *guyanensis *	M	30	10	P	1	32 × 21
MHOM/BR/11/IM5775	*L. guyanensis *	M	24	21	P	4	8 × 5
MHOM/BR/11/IM5828	*L. guyanensis *	M	43	150	P	1	11 × 8
MHOM/BR/11/IM5833	*L. guyanensis *	F	21	30	P	1	70 × 70
MHOM/BR/11/IM5869	*L. guyanensis *	M	24	60	P	8	60 × 60
MHOM/BR/11/IM5875	*L. guyanensis *	M	49	30	P	1	11 × 7
MHOM/BR/11/IM5894	*L. guyanensis *	M	27	30	S	1	20 × 12
MHOM/BR/11/IM5950	*L*. *amazonensis *	F	30	30	P	1	25 × 26
MHOM/BR/11/IM5955	*L. amazonensis *	F	29	15	P	1	60 × 90
MHOM/BR/11/IM5962	*Leishmania* sp.	M	30	60	S	2	6 × 5
MHOM/BR/11/IM5969	*Leishmania* sp.	F	30	15	S	1	10 × 7
MHOM/BR/11/IM5976	*L. guyanensis *	F	18	21	P	1	17 × 13
MHOM/BR/11/IM5985	*L*. *guyanensis *	F	25	15	P	1	12 × 12
MHOM/BR/12/IM6006	*Leishmania* sp.	M	33	180	S	1	ND
MHOM/BR/12/IM6016	*Leishmania* sp.	F	30	120	S	04	11 × 11
MHOM/BR/12/IM6025	*Leishmania* sp.	M	31	7	S	1	1 × 1
MHOM/BR/12/IM6034	*L*. *amazonensis *	M	42	21	P	1	29 × 15
MHOM/BR/12/IM6038	*Leishmania* sp.	F	48	30	S	1	17 × 17

*All biological material was collected by healthcare professionals at the Joint Health Unit Thomé de Medeiros Raposo and the Basic Health Unit Manoel Rumão, Municipality of Rio Preto da Eva.

^1^Designations: host [M = Mammalia: HOM = *Homo sapiens *]/country of origin/year of isolation/original code used by INPA; ^2^stock identification was established by enzyme electrophoresis; ^3^length of infection indicates the approximate time in days when patients recalled appearance of symptoms or lesion; number of lesion = number of ulcerated skin lesion in a patient; infection status refers to either primary (P) or secondary (S) infection; lesion size = measurements (*L* × *W*) of lesions. ND: not determined.

## References

[1] Alvar J, Vélez ID, Bern C (2012). Leishmaniasis worldwide and global estimates of its incidence. *PLoS ONE*.

[2] Silveira FT, Lainson R, Corbett CEP (2004). Clinical and immunopathological spectrum of American cutaneous leishmaniasis with special reference to the disease in Amazonian Brazil—a review. *Memorias do Instituto Oswaldo Cruz*.

[3] SINAN/SVS/MS (2012). *Boletim Eletrônico Epidemiológigo*.

[4] Silveira FT, Ishikawa EAY, de Souza AAA, Lainson R (2002). An outbreak of cutaneous leishmaniasis among soldiers in Belém, Pará State, Brazil, caused by *Leishmania (Viannia) lindenbergi* n. sp. A new leishmanial parasite of man in the Amazon region. *Parasite*.

[5] Naiff MF, Cupolillo E, Naiff RD, Momen H, Barret TV, Grimaldi JRG (1999). Leishmaniose tegumentar americanana Amazônia: distribuição geográfica dos agentes etiológicos naregião. *Revista da Sociedade Brasileira de Medicina Tropical*.

[6] Figueira LDP, Zanotti M, Pinheiro FG, Franco AMR (2008). Isoenzymatic characterization of human isolates of *Leishmania* sp (Kinetoplastida: Trypanosomatidae) from the municipalities of Rio Preto da Eva and Manaus, State of Amazonas. *Revista da Sociedade Brasileira de Medicina Tropical*.

[7] Mauël J (2002). Vaccination against Leishmania infections. *Current Drug Targets: Immune, Endocrine and Metabolic Disorders*.

[8] Silveira FT, Mülher SR, Souza AA, Lainson R, Gomes CM, Laurent MD (2008). Revisão sobre a patogenia da Leishmaniose Tegumentar Americana na Amazônia, com ênfase à doença causada por *Leishmania (V.) braziliensis e Leishmania (L.) amazonensis*. *Revista Paraense de Medicina*.

[9] Gontijo B, Carvalho MLR (2003). Leishmaniose Tegumentar Americana. *Revista da Sociedade Brasileira de Medicina Tropical*.

[10] Prates DB, Araújo-Santos T, Luz NF (2011). Lutzomyia longipalpis saliva drives apoptosis and enhances parasite burden in neutrophils. *Journal of Leukocyte Biology*.

[11] Sacks D, Noben-Trauth N (2002). The immunology of susceptibility and resistance to Leishmania major in mice. *Nature Reviews Immunology*.

[12] Wanasen N, Soong L (2008). L-arginine metabolism and its impact on host immunity against Leishmania infection. *Immunologic Research*.

[13] Oliveira RA, Lima CG, Mota RM (2012). Renal function evaluation in patients with American Cutaneous Leishmaniasis after specific treatment with pentavalent antimonial. *BMC Nephrology*.

[14] Romero GAS, de Farias Guerra MV, Paes MG, de Oliveira Macêdo V (2001). Comparison of cutaneous leishmaniasis due to *Leishmania* (*Viannia*) *braziliensis* and *Leishmania* (*V*.) *guyanensis* in Brazil: Therapeutic response to meglumine antimoniate. *American Journal of Tropical Medicine and Hygiene*.

[15] Arevalo J, Ramirez L, Adaui V (2007). Influence of *Leishmania* (*Viannia*) species on the response to antimonial treatment in patients with American tegumentary leishmaniasis. *The Journal of Infectious Diseases*.

[16] Ministério da Saúde (Brasil) (2007). *Manual de Vigilância da Leishmaniose Tegumentar Americana*.

[17] Novy FG, MacNeal WJ (1904). On the cultivation of *Trypanosoma brucei*. *The Journal of Infectious Diseases*.

[18] Nicolle GL (1908). Culture du parasite du Bouton d'Orient. *Comptes Rendus de l'Académie des Sciences*.

[19] Cupolillo E, Grimaldi G, Momen H (1994). A general classification of new world Leishmania using numerical zymotaxonomy. *The American Journal of Tropical Medicine and Hygiene*.

[20] Alexander WS, Hilton DJ (2004). The role of suppressors of cytokine signaling (SOCS) proteins in regulation of the immune response. *Annual Review of Immunology*.

[21] Martinez FO, Helming L, Gordon S (2009). Alternative activation of macrophages: an immunologic functional perspective. *Annual Review of Immunology*.

[22] Bogdan C, Röllinghoff M, Diefenbach A (2000). The role of nitric oxide in innate immunity. *Immunological Reviews*.

[23] Rautajoki KJ, Kyläniemi MK, Raghav SK, Rao K, Lahesmaa R (2008). An insight into molecular mechanisms of human T helper cell differentiation. *Annals of Medicine*.

[24] Kaiko GE, Horvat JC, Beagley KW, Hansbro PM (2008). Immunological decision-making: how does the immune system decide to mount a helper T-cell response?. *Immunology*.

[25] Ribeiro-de-Jesus A, Almeida RP, Lessa H, Bacellar O, Carvalho EM (1998). Cytokine profile and pathology in human leishmaniasis. *Brazilian Journal of Medical and Biological Research*.

[26] Reithinger R, Dujardin J-C, Louzir H, Pirmez C, Alexander B, Brooker S (2007). Cutaneous leishmaniasis. *The Lancet Infectious Diseases*.

[27] Nylén S, Gautam S (2010). Immunological perspectives of leishmaniasis. *Journal of Global Infectious Diseases*.

[28] Murray HW, Lu CM, Mauze S (2002). Interleukin-10 (IL-10) in experimental visceral leishmaniasis and IL-10 receptor blockade as immunotherapy. *Infection and Immunity*.

[29] Trinchieri G (2007). Interleukin-10 production by effector T cells: Th1 cells show self control. *Journal of Experimental Medicine*.

[30] Bacellar O, Lessa H, Schriefer A (2002). Up-regulation of Th1-type responses in mucosal leishmaniasis patients. *Infection and Immunity*.

[31] Degrossoli A, Arrais-Silva WW, Colhone MC, Gadelha FR, Joazeiro PP, Giorgio S (2011). The influence of low oxygen on macrophage response to *Leishmania* infection. *Scandinavian Journal of Immunology*.

[32] Reiner SL, Locksley RM (1995). The regulation of immunity to Leishmania major. *Annual Review of Immunology*.

[33] Liew FY, Wei X-Q, Proudfoot L (1997). Cytokines and nitric oxide as effector molecules against parasitic infections. *Philosophical Transactions of the Royal Society B: Biological Sciences*.

[34] Pinheiro RO, Rossi-Bergmann B (2007). Interferon-gamma is required for the late but not early control of *Leishmania amazonensis* infection in C57Bl/6 mice. *Memorias do Instituto Oswaldo Cruz*.

[35] Kima PE, Soong L (2013). Interferon gamma in leishmaniasis. *Frontiers in Immunology*.

[36] Latifynia A, Khamesipour A, Bokaie S, Khansari N (2012). Antioxidants and proinflamatory cytokines in the sera of patients with cutaneous leishmaniasis. *Iranian Journal of Immunology*.

[37] Kolls JK, Lindén A (2004). Interleukin-17 family members and inflammation. *Immunity*.

[38] Kostka SL, Dinges S, Griewank K, Iwakura Y, Udey MC, Von Stebut E (2009). IL-17 promotes progression of cutaneous leishmaniasis in susceptible mice. *The Journal of Immunology*.

[39] Lee W-W, Kang SW, Choi J (2010). Regulating human Th17 cells via differential expression of IL-1 receptor. *Blood*.

[40] Bacallar O, Faria D, Nascimento M (2009). Interleukin 17 production among patients with American cutaneous leishmaniasis. *Journal of Infectious Diseases*.

[41] Bueno LL, Morais CG, Lacerda MV, Fujiwara RT, Braga ÉM (2012). Interleukin-17 producing T helper cells are increased during natural *Plasmodium vivax* infection. *Acta Tropica*.

[42] Boaventura VS, Santos CS, Cardoso CR (2010). Human mucosal leishmaniasis: neutrophils infiltrate areas of tissue damage that express high levels of Th17-related cytokines. *European Journal of Immunology*.

[43] Miralles GD, Stoeckle MY, McDermott DF, Finkelman FD, Murray HW (1994). Th1 and Th2 cell-associated cytokines in experimental visceral Leishmaniasis. *Infection and Immunity*.

[44] Hurdayal R, Brombacher F (2014). The role of IL-4 and IL-13 in cutaneous Leishmaniasis. *Immunology Letters*.

[45] Siewe L, Bollati-Fogolin M, Wickenhauser C, Krieg T, Müller W, Roers A (2006). Interleukin-10 derived from macrophages and/or neutrophils regulates the inflammatory response to LPS but not the response to CpG DNA. *European Journal of Immunology*.

[46] Sabat R (2010). IL-10 family of cytokines. *Cytokine and Growth Factor Reviews*.

[47] Mendez S, Reckling SK, Piccirillo CA, Sacks D, Belkaid Y (2004). Role for CD4^+^ CD25^+^ regulatory T cells in reactivation of persistent Leishmaniasis and control of concomitant immunity. *Journal of Experimental Medicine*.

[48] Belkaid Y (2007). Regulatory T cells and infection: a dangerous necessity. *Nature Reviews Immunology*.

[49] Bittar RC, Nogueira RS, Vieira-Gonçalves R (2007). T-cell responses associated with resistance to Leishmania infection in individuals from endemic areas for Leishmania (Viannia) braziliensis. *Memorias do Instituto Oswaldo Cruz*.

[50] Belkaid Y, Hoffmann KF, Mendez S (2001). The role of interleukin (IL)-10 in the persistence of Leishmania major in the skin after healing and the therapeutic potential of anti-IL-10 receptor antibody for sterile cure. *Journal of Experimental Medicine*.

[51] Kaye EP, Scott P (2011). Leishmaniasis: complexity at the host-pathogen interface. *Nature Reviews Microbiology*.

[52] Reis LC, Brito MEF, Souza MA (2009). Cellular immune response profile in patients with american tegumentary leishmaniasis prior and post chemotherapy treatment. *Journal of Clinical Laboratory Analysis*.

[53] Scott P (2011). Leishmania—a parasitized parasite. *The New England Journal of Medicine*.

